# Association of weight-adjusted waist index with all-cause and cause-specific mortality among cancer survivors: a cohort study of the NHANES 1999-2018

**DOI:** 10.3389/fendo.2024.1422071

**Published:** 2024-11-07

**Authors:** Shi Li, Jing Jin, Wenshun Zhang, Ying Cao, Haiyun Qin, Jianguang Wang, Jiaxiang Yu, Wenping Wang

**Affiliations:** ^1^ Liaoning University of Traditional Chinese Medicine, Shenyang, China; ^2^ The Second Affiliated Hospital of Liaoning University of Traditional Chinese Medicine, Shenyang, China; ^3^ Affiliated Hospital of Liaoning University of Traditional Chinese Medicine, Shenyang, China

**Keywords:** weight-adjusted waist index, cancer, all-cause mortality, obesity, cardiovascular mortality

## Abstract

**Background:**

Obesity is becoming more widely acknowledged as a chronic illness that raises the risk of oncogenesis. This inquiry aimed to look into the correlation between cancer patient mortality and obesity, as measured by the weight-adjusted waist index (WWI).

**Methods:**

We used continuous data from the National Health and Nutrition Examination Survey (NHANES) from 1999 to 2018 as the benchmark, with a follow-up validity period of December 31, 2019. First, we assessed the correlation between WWI and the all-cause and cause-specific execution of cancer sufferers using multivariable Cox proportional hazards models. Second, a smoothed curve fit was utilized to examine the relationship between WWI and both cause-specific and all-cause mortality in cancer patients. Lastly, we analyzed the relationship between WWI and both cause-specific and all-cause mortality in cancer patients, to find out if this link held across the population subgroup evaluation and impact analyses were used as well during the last step.

**Results:**

With a median follow-up of 87.8 months, 1,547 (34.7%) of the 4,463 cancer patients had deceased. Among them, 508 (11.4%) succumbed to cancer, while 322 (7.2%) passed away due to cardiovascular disease. Multivariate Cox proportional hazards model of mortality among cancer patients revealed an all-cause mortality hazard ratio [HR=1.13; 95% CI (1.04, 1.23)] and cardiovascular mortality [HR=1.39; 95% CI (1.16, 1.67)]. Furthermore, for each unit increase in WWI, all-cause mortality was significantly higher in male cancer survivors than in female cancer survivors.

**Conclusions:**

Our study reveals substantial correlations between WWI and all-cause and cardiovascular mortality in US cancer survivors, helping to identify cancer survivors at higher risk of death and thus potentially guiding targeted interventions.

## 1 Introduction

Cancer constitutes a significant public health challenge with profound implications for human well-being. The World Health Organization (WHO) and the International Agency for Research on Cancer (IARC) released the most current information on the worldwide incidence of cancer in 2020. This information showed that there were about 19.29 million new cancer cases recorded globally. Forecasts show a concerning probable increase to 28.4 million episodes by 2040, which would be a significant rise of 47% from the numbers of 2020 (1). Therefore, identifying factors that may be changed or prevented is essential to reduce the incidence and death rate related to cancer.

As a significant public health concern, obesity now affects almost 35% of adult Americans. This condition significantly raises the risk of acquiring different kinds of cancer, including those of the breast, uterine, prostate, and digestive system, and is closely associated with a poor prognosis (2, 3). Furthermore, severely obese individuals, diagnosed with cancer, face an elevated risk of mortality (4). A substantial quantity of information has been acquired about the relationship between obesity, increased cancer rates, and associated mortality throughout the last 20 years. The percentage of cancer-related deaths that are directly linked to obesity is 14% for males and 20% for females (5).

Waist circumference (WC) and Body Mass Index (BMI) are widely accepted as the most accurate metrics in the field of evaluating obesity. However, their reliability is not absolute, as they can potentially overlook fluctuations in body composition and disregard differences in body size (6, 7). The usefulness of body composition and fatty tissue distribution, in more precisely defining metabolic features, has been pointed to by recent studies (8, 9). WWI is a novel obesity metric that combines the advantages of both metrics by integrating body weight and waist circumference and eliminating the unclear relationship between WC and BMI (10). WWI incorporates an individual’s weight into the evaluation of central obesity, enabling a more holistic appraisal of obesity-related health risks, and overcoming the intrinsic limitations of the traditional measures. It does this by offering a nuanced perspective that encompasses both obesity and body shape (11, 12).

The mechanisms of cancer morbidity and mortality are composite, and being obese is regarded as one of the primary risk factors for cancer incidence (13). Although WWI is useful in identifying different hazards associated with cardiovascular disease, few studies have examined whether it is also associated with all-cause mortality as well as cardiovascular mortality among cancer patients. Consequently, our goal was to use cohort analyses and data from the 1999–2018 NHANES to look into the connection between these variables.

## 2 Methods

### 2.1 Study population

**Figure 1:**
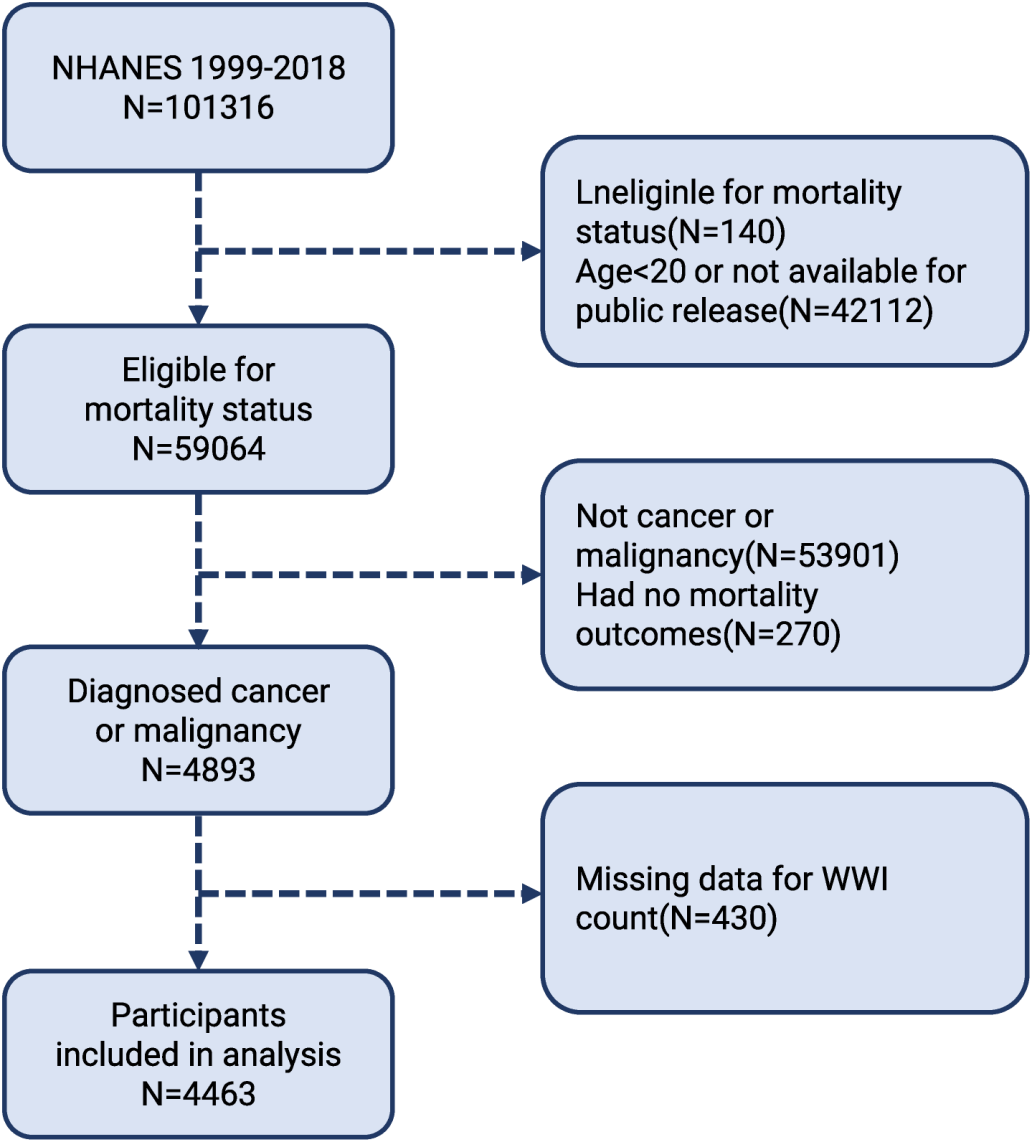
Flow chart of participants selection. NHANES, National Health and Nutrition Examination Survey.

The purpose of the cohort study of the NHANES is to gather data on nutrition and health from the US populace using an unbiased survey. The data were collected by laboratory testing, home-based structured interviews, and thorough physicals at mobile centers, using a multi-stage probabilistic sampling technique. The National Center for Health Statistics (NCHS) Ethics Review Committee authorized the NHANES methodology, and each participant gave their informed consent. We downloaded data for 1999-2018 from the NHANES website. www.cdc.gov/nchs/nhanes/indexhtm), covering ten survey cycles. A total of 101,316 participants were screened in the NHANES cohort during this period. Given the relationship between oestrogenic effects affecting body fat distribution around puberty, we need to carefully consider sample selection when conducting related studies or data analyses and to minimize confounding by physiological changes during puberty, therefore, we excluded individuals who were under the age of twenty (20) (n = 42,112). Furthermore, the examination of non-death outcomes (n = 275) did not include people without cancer or malignancy (n = 53,901). We also excluded those without a WWI count (n = 430). As a result, the study included 4,463 participants (**Figure 1**).

### 2.2 Cancer status

Data in the health condition section of NHANES is gathered from self-reported interviews. A cancer diagnosis was set up using a pair of questions: 1. “Have you ever been told by a doctor or other health professional that you had cancer or a malignancy of any kind? “ 2. “What kind of cancer was it and when it was diagnosed?”. These inquiries were recorded in the participants’ homes by skilled interviewers, by applying computer-assisted personal interviewing (CAPI) technology (14). Integrated consistency checks are a component of CAPI systems that help to decrease data entry errors.

### 2.3 Definition of WWI

The WWI is a neoteric method of evaluating obesity. Higher WWI scores are connected to adiposity levels. This index considers a person’s weight and provides information about central obesity. The computation involves dividing the waist circumference (WC) by the square root of the weight (10). Anthropometric measures are performed by on-site medical professionals, and data is thoroughly recorded by qualified individuals, to guarantee accuracy. During this process, participants, in examination clothes, stand on a digital scale barefooted, with their faces turned to the front and their hands clasped in front of their bodies (15). A horizontal line was drawn above the highest lateral margin of the right ilium, the midline of the right armpit was drawn, and a tape measure was placed at the intersection of the two lines to determine waist circumference (16). In the research we’ve conducted, WWI is regarded as a sensitive condition.

### 2.4 Measurements of mortality

As of December 31, 2019, the public can receive monitoring mortality data from the NHANES Mobile Inspection Centre (MEC) via the publicly available Linked Mortality Files (LMF). Information about mortality rates and probable reasons for death can be found in this database. Using the tenth version of the International Classification of Diseases (ICD-10), cause-specific mortality was calculated. People who were ineligible for public release or who did not have enough identifiable information were not included in this study.

### 2.5 Covariates

Based on the relevant literature, we considered several potential confounders that may influence the association between WWI and the risk of all-cause and cardiovascular mortality in cancer patients. These covariates included age, sex, race, BMI, education, smoking status, physical activity, diabetes, high blood pressure, coronary heart disease, income-to-poverty ratio (PIR), high-density lipoprotein cholesterol/low-density lipoprotein cholesterol (HDL/LDL). Race was classified as non-Hispanic white, non-Hispanic Black, Mexican American, and other race/multiracial. Smoking status was classified as never-smoker, and whether or not they had smoked at least 100 cigarettes in their lifetime. Physical activity was established by a question: “Does your work involve moderate-intensity activity that causes small increases in breathing or heart rate such as brisk walking or carrying light loads for at least 10 minutes continuously?” A diagnosis of high blood pressure is established by a question: “Have you ever been told by a doctor or other health professional that you had hypertension, also called high blood pressure?” A diagnosis of diabetes is established by a question: “Other than during pregnancy, have you ever been told by a doctor or health professional that you have diabetes or sugar diabetes?”

### 2.6 Statistical analysis

This study clung to the CDC’s guidelines for statistical analysis, and participant characteristics were assessed utilizing multivariable Cox proportional hazards models based on the WWI quartile. Excluding covariates with multicollinearity by Variance Inflation Factor (VIF) test. This study examined the relationship between WWI and cardiovascular mortality and all-cause mortality in cancer survivors using three different multivariate Cox proportional hazards models. Model 1 was not modified to account for variables. Model 2 possessed BMI, race, sex, and age modifications. Model 3 considered novel variables such as BMI, age, sex, race, PIR, HDL/LDL, BMI, education, smoking, exercise, diabetes, high blood pressure, and coronary heart disease. The WWI score was divided into quartiles, then trend tests were run, to look into any potential correlations between the WWI score, and cardiovascular and all-cause deaths among cancer patients. The effects of the main covariates on all-cause mortality and cardiovascular mortality were derived by univariate analysis. A subgroup analysis was performed to inquire into any differences between several clinical variables and demographics, including age, sex, race, BMI, PIR, education, smoking, diabetes, high blood pressure, and coronary heart disease. This process aimed to evaluate consistency across various subgroups. The potential non-linear interaction between death outcomes and WWI was examined through the use of smooth curve fitting (17). All of the analyses were regulated using either Empowerstats (version 5.0) or R (version 4.2). A value of P <0.05 on two sides was accepted as statistically significant.

## 3 Results

### 3.1 Baseline characteristics

After applying the exclusion criteria, in total, 4,463 cancer survivors over the age of 20 were evaluated in this study’s evaluation of data from NHANES, which ran from 1999 to 2018 (**Figure 1**). Their baseline characteristics are summarised in **Table 1** and stratified by WWI quartiles. The interquartile method involves arranging the values from smallest to largest and dividing the total into four equal parts, which in turn analyses the trend of the variable. The mean age of the included patients was 65.34 years and 52.61% of the patients were female. Among the participants, the WWI averaged 11.3 with the following quartiles: quartile 1 (7.90–10.85); quartile 2 (10.85–11.38); quartile 3 (11.38–11.89); quartile 4 (11.38–11.89). The demographics of people who scored higher on WWI were female, older, non-Hispanic White, high school graduates, less active, and more likely to have diabetes and elevated blood pressure contrasted with those who scored in the lowest quartile. Furthermore, **Table 1** shows a correlation between decreased HDL/LDL and PIR levels, and higher WWI.

**Table 1:** Basic characteristics of participants by weight-adjusted waist index quartile.

Characteristics	weight-adjusted waist index quartile (cm/√kg)				*P*-value
	Q1(<10.85) N= 1116	Q2 (10.85-11.3) N=1115	Q3 (11.38-11.89) N=1116	Q4 (>11.89) N=1116	
Age (years)	57.59 ± 15.79	65.56 ± 13.83	68.39 ± 12.68	69.82 ± 12.14	<0.001
Sex, (%)					<0.001
Male	45.25	53.81	53.14	37.37	
Female	54.75	46.19	46.86	62.63	
Race/ethnicity, (%)					<0.001
Non-Hispanic White	67.11	69.24	69.89	71.06	
Non-Hispanic Black	19.44	15.52	12.37	8.24	
Mexican American	4.57	6.46	7.71	9.95	
Other race/multiracial	8.87	8.79	10.04	10.75	
Education level, (%)					<0.001
Less than high school	13.99	21.61	23.07	31.57	
High school	21.88	23.41	23.25	25.47	
More than high school	64.13	54.98	53.68	42.96	
Smoking, (%)					0.336
Ever	54.49	54.80	57.94	56.27	
Never	45.51	45.20	42.06	43.73	
Exercise over the past 30 days					<0.001
Yes	45.16	44.57	39.61	33.15	
No	54.84	55.43	60.39	66.85	
Coronary heart disease, (%)					<0.001
Yes	5.22	10.67	11.90	11.56	
No	94.78	89.33	88.10	88.44	
Stroke, (%)					<0.001
Yes	4.49	7.46	8.16	11.39	
No	95.51	92.54	91.84	88.61	
Diabetes, (%)					<0.001
Yes	9.32	18.74	24.28	34.41	
No	90.68	81.26	75.72	65.59	
High blood pressure, (%)					<0.001
Yes	40.59	54.63	59.01	66.34	
No	59.41	45.37	40.99	33.66	
BMI (kg/m^2^)	25.53 ± 5.27	27.81 ± 5.24	29.49 ± 5.71	32.01 ± 6.91	<0.001
Waist circumference (cm)	88.72 ± 11.20	98.61 ± 11.25	104.88 ± 12.17	112.65 ± 14.62	<0.001
Weight (kg)	73.93 ± 17.09	79.53 ± 18.12	82.39 ± 19.06	84.57 ± 21.81	<0.001
Height(cm)	169.92 ± 9.85	168.65 ± 9.56	166.74 ± 9.19	161.98 ± 9.31	<0.001
PIR	3.12 ± 1.63	2.89 ± 1.53	2.68 ± 1.46	2.33 ± 1.38	<0.001
HDL/LDL	0.55 ± 0.25	0.51 ± 0.22	0.48 ± 0.19	0.49 ± 0.19	<0.001
Status, n (%)					<0.001
Alive	74.64	65.11	60.75	60.84	
Death	25.36	34.89	39.25	39.16	

Data are expressed as mean ± stand error or frequency (percentage).

Q, quartile; PIR, Ratio of family income to poverty; BMI, body mass index; HDL/LDL, high-density lipoprotein cholesterol/low-density lipoprotein cholesterol.

### 3.2 Association between WWI and mortality outcomes

The number of individual months of work from the completion of the questioning divided by the period of the fatality or the closure of the life expectancy period was used to calculate the follow-up period. After a median follow-up period of 87.8 months, the all-cause mortality rate among cancer patients in the United States was 34.66% (n = 1547), with cardiovascular mortality accounting for 7.21% (n = 322). There was a strong positive correlation between WWI and all-cause mortality, according to both the crude model [HR=1.47; 95% CI (1.38, 1.57)] and the partially adjusted model [HR=1.27; 95%CI (1.17, 1.38)]. This strong link held statistical significance even after full adjustment, with a 13% increase in all-cause mortality for every unit increase in WWI [HR=1.13; 95%CI (1.04, 1.23)]. Furthermore, we found a substantial association between a high risk of cardiovascular death and WWI. Following full correction, there was a substantial increase in the risk of cardiovascular death with growing WWI: HR= 1.39; 95% CI (1.16, 1.67). When the adjusted quartiles were analyzed, we still observed a significant upward trend in cardiovascular mortality. Those included in the top WWI quartile (HR=1.88; 95% CI [1.24, 2.86]) had a prevalence that was 65% higher than those in the lowest quartile. The risk of developing CVD was higher for those in the highest quartile of WWI compared to those in the lowest quartile (**Table 2**). With non-adjusted models [HR=4.41; 95% CI (3.02, 6.44)], Model II [HR=2.20; 95% CI (1.46, 3.32)], and Model III [HR=1.88; 95% CI (1.24, 2.86)].

**Table 2:** The associations between WWI(cm/√kg) and mortality.

	Q1	Q2	Q3	Q4	P for Trend	WWI(continuous)
All-cause Mortality (1547/4463)						
Model 1 [HR (95% CI)]	1.00	1.60 (1.37, 1.86)	1.96 (1.68, 2.27)	1.09 (0.92, 1.29)	< 0.001	1.47 (1.38, 1.57)
Model 2 [HR (95% CI)]	1.00	1.09 (0.93, 1.28)	1.24 (1.05, 1.45)	1.51 (1.28, 1.79)	< 0.001	1.27 (1.17, 1.38)
Model 3 [HR (95% CI)]	1.00	1.03 (0.88, 1.21)	1.09 (0.93, 1.28)	1.25 (1.05, 1.49)	0.0028	1.13 (1.04, 1.23)
CVD Mortality (322/4463)						
Model 1 [HR (95% CI)]	1.00	2.50 (1.68, 3.71)	3.54 (2.42, 5.19)	4.41 (3.02, 6.44)	< 0.001	1.92 (1.66, 2.21)
Model 2 [HR (95% CI)]	1.00	1.39 (0.93, 2.09)	1.67 (1.12, 2.49)	2.20 (1.46, 3.32)	< 0.001	1.55 (1.29, 1.85)
Model 3 [HR (95% CI)]	1.00	1.31 (0.87, 1.97)	1.45 (0.97, 2.17)	1.88 (1.24, 2.86)	0.0005	1.39 (1.16, 1.67)

Model 1: no covariates were adjusted. Model 2: age, sex, race, and BMI were adjusted. Model 3: age, sex, race, BMI, education, smoking, activity, diabetes, high blood pressure, coronary heart disease, PIR, HDL/LDL were adjusted.

Q, quartile; PIR, Ratio of family income to poverty; BMI, body mass index; HDL/LDL, high-density lipoprotein cholesterol/low-density lipoprotein cholesterol.

### 3.3 Smooth curve fitting

**Figure 2:**
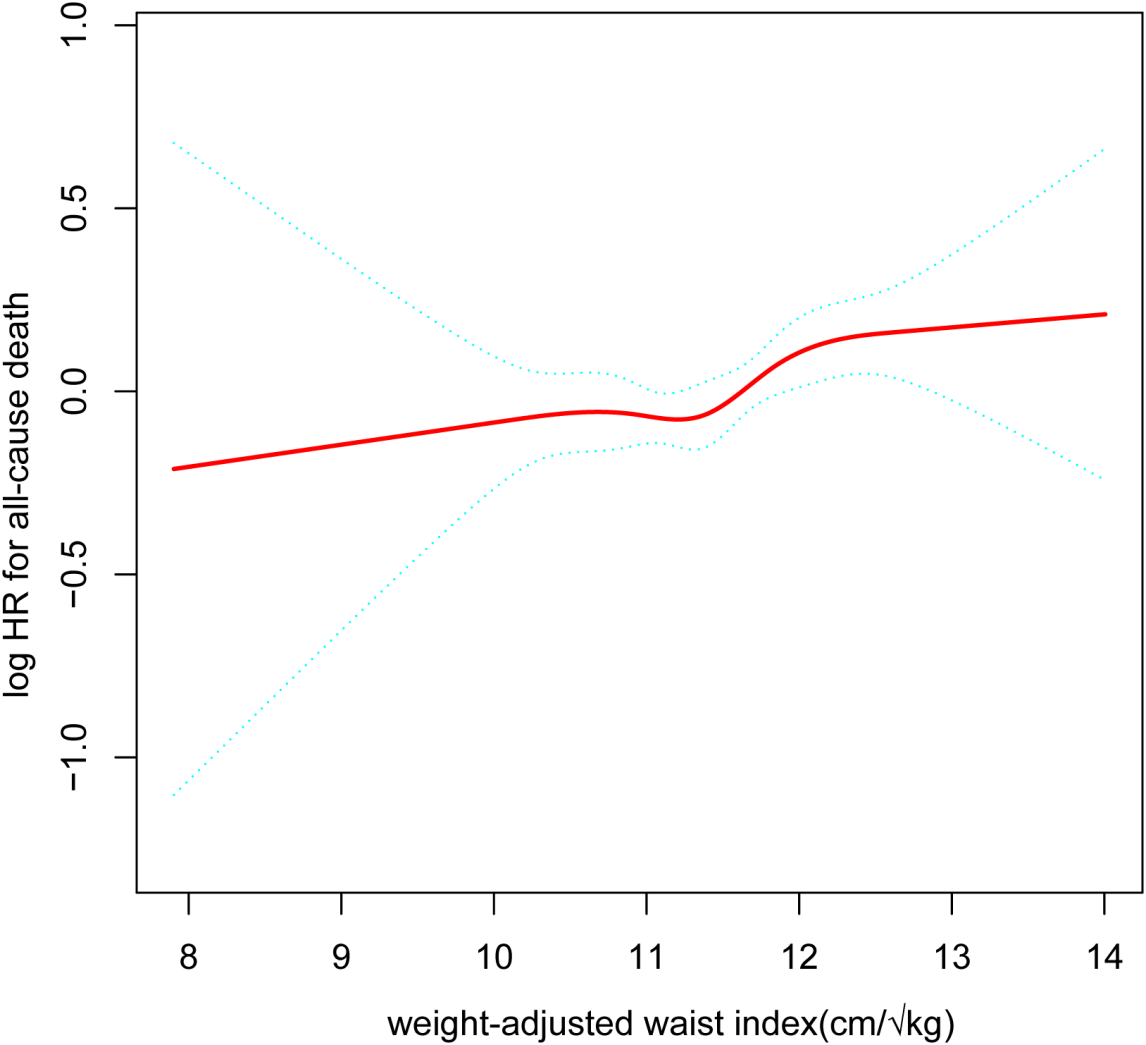
The nonlinear relationship between cancer patients’ all-cause death and WWI. The smooth curve fit between the variables is represented by the solid red line. The 95% confidence interval from the fit is shown by blue bands. (WWI, weight-adjusted waist index).

**Figure 3:**
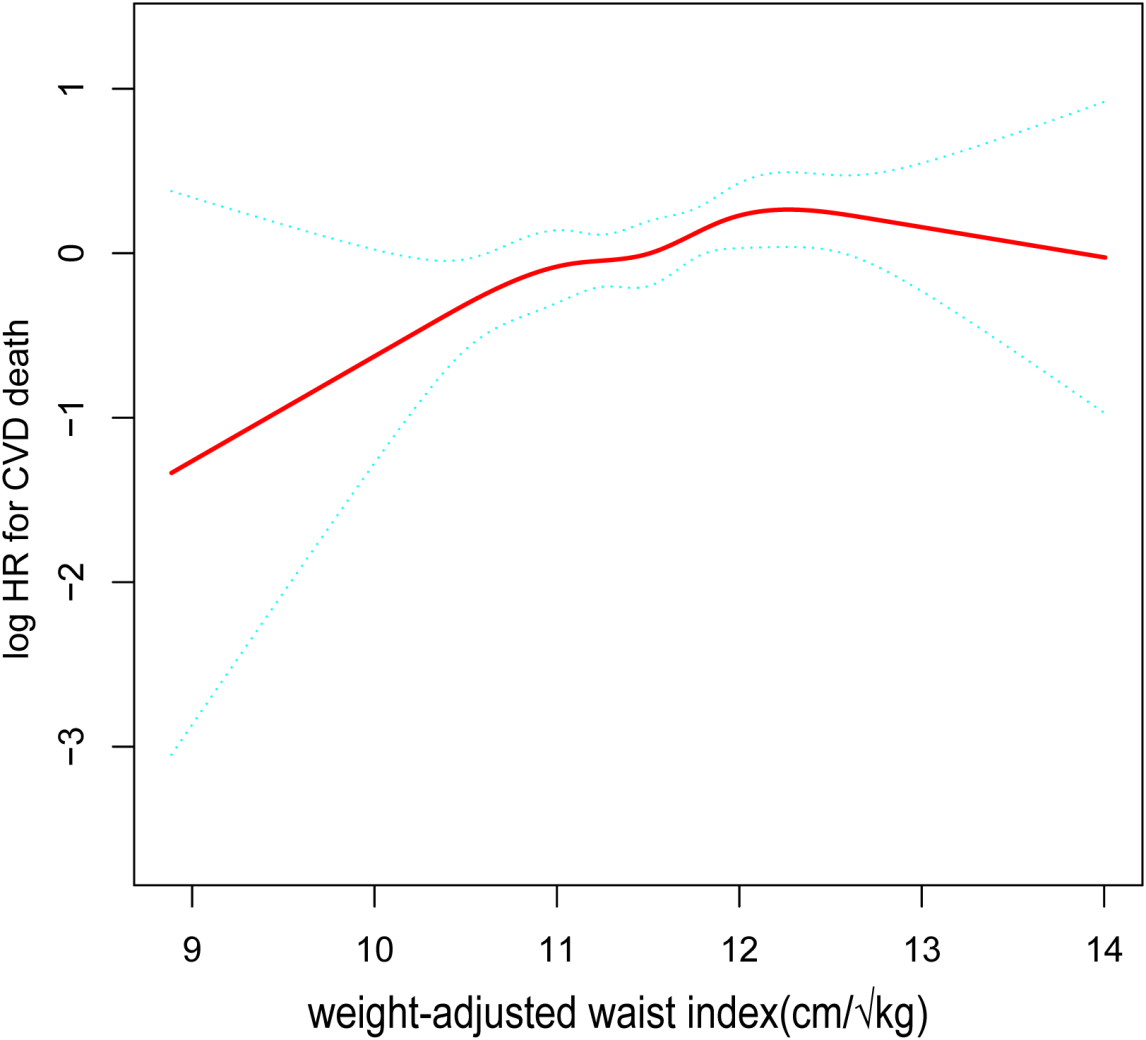
The nonlinear relationship between cardiovascular mortality in cancer patients and WWI. The smooth curve fit between the variables is shown by the solid red line. The 95% confidence interval derived from the fit is shown by blue bands. (WWI, weight-adjusted waist index).

After accounting for all variables, we produced a smooth curve that illustrates the connection between WWI and the cardiovascular and all-cause deaths of cancer patients. Our analysis revealed a nonlinear positive correlation in this patient population between WWI and all-cause mortality, as well as cardiovascular disease (**Figure 2**, **Figure 3**). The higher the WWI in cancer survivors, the higher the all-cause mortality and cardiovascular mortality.

### 3.4 Univariate analysis

We conducted univariate analyses of the associations of key covariates with all-cause mortality and cardiovascular mortality in cancer patients. The results found that cancer survivors who were male, older, in poorer family conditions, less physically active, diabetic, and coronary heart disease had higher all-cause mortality and cardiovascular mortality. In addition, cardiovascular mortality was higher in cancer patients with hypertension, and all-cause mortality was higher in cancer patients with smoking habits (**Table 3**).

**Table 3:** Results of the association of covariates with all-cause mortality and cardiovascular mortality in cancer survivors.

Covariates	All-cause Mortality [HR (95%CI)]	CVD Mortality [HR (95%CI)]
Age	1.07 (1.07, 1.08)	1.12 (1.10, 1.14)
Sex		
Male	1.38 (1.24, 1.54)	1.53 (1.20, 1.95)
Female	1.0	1.0
Race/ethnicity		
Non-Hispanic White	1.0	1.0
Non-Hispanic Black	1.17 (1.00, 1.37)	1.04 (0.72, 1.51)
Mexican American	0.78 (0.62, 1.00)	0.44 (0.22, 0.90)
Other race/multiracial	0.74 (0.58, 0.95)	0.32 (0.14, 0.73)
Education level		
Less than high school	1.0	1.0
High school	0.95 (0.83, 1.09)	1.05 (0.78, 1.43)
More than high school	0.88 (0.78, 1.01)	1.05 (0.79, 1.40)
Smoking		
Ever	1.32 (1.19, 1.47)	1.12 (0.89, 1.41)
Never	1.0	1.0
Exercise over the past 30 days		
Yes	0.74 (0.67, 0.83)	0.77 (0.61, 0.97)
No	1.0	1.0
Coronary heart disease,		
Yes	1.23 (1.07, 1.42)	1.85 (1.42, 2.42)
No	1.0	1.0
Diabetes		
Yes	1.27 (1.13, 1.43)	1.46 (1.13, 1.88)
No	1.0	1.0
High blood pressure		
Yes	1.10 (0.99, 1.23)	1.31 (1.03, 1.68)
No	1.0	1.0
BMI	0.96(0.88,1.04)	1.00 (0.98, 1.02)
PIR	0.83 (0.80, 0.87)	0.86 (0.79, 0.93)
HDL/LDL	1.21 (0.96, 1.53)	0.88 (0.51, 1.51)

PIR, Ratio of family income to poverty; BMI, body mass index; HDL/LDL, high-density lipoprotein cholesterol/low-density lipoprotein cholesterol.

### 3.5 Subgroup analyses for WWI and mortality

**Figure 4:**
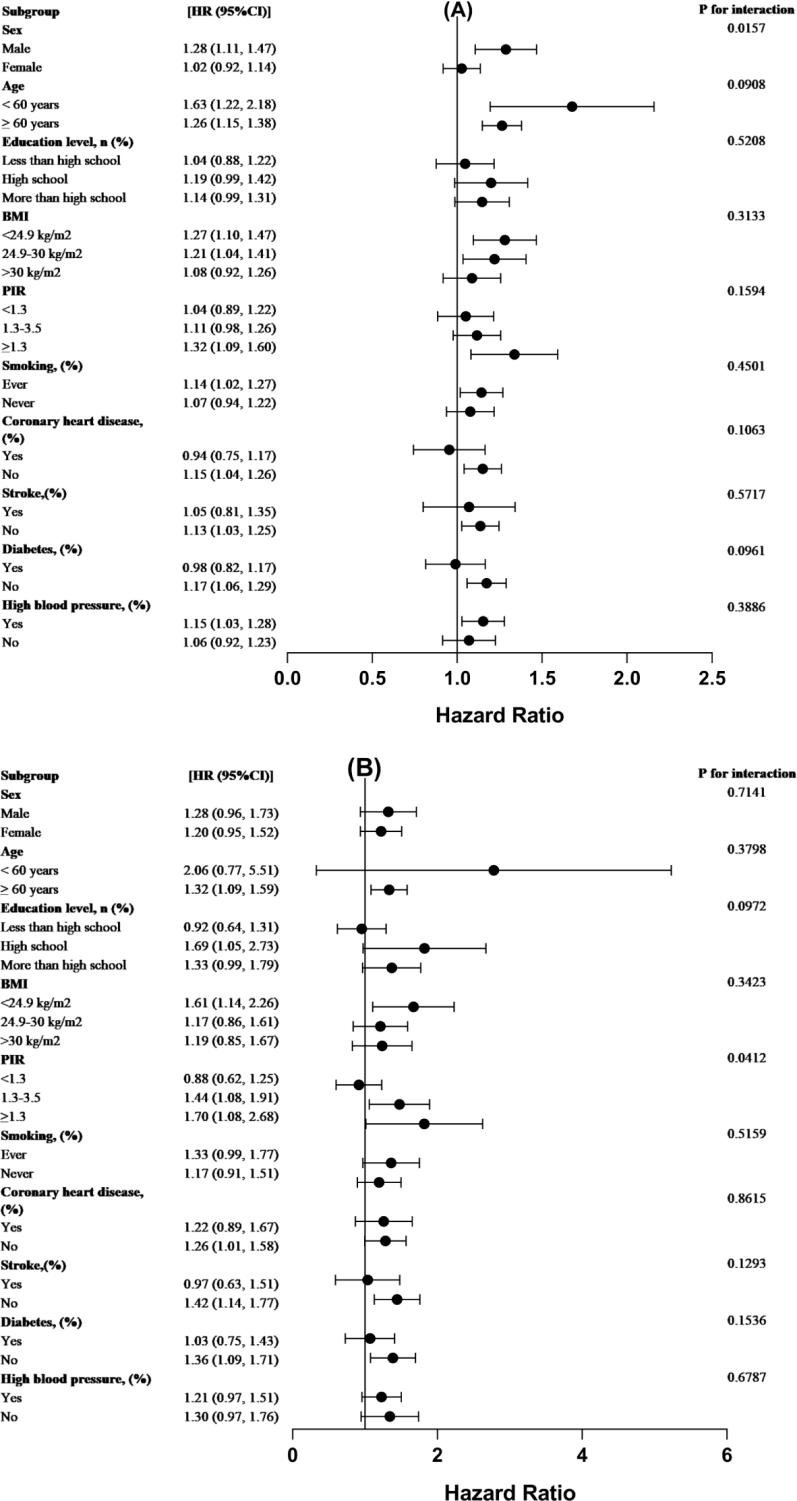
For the population-wide stratified study, forest plots were used. The WWI(cm/√kg association between) analysis for **(A)** all-cause mortality and **(B)** CVD mortality among cancer patients, stratified by demographic variables, is displayed in forest plot form. The analysis was modified to account for the following factors: education, smoking, diabetes, high blood pressure, coronary heart disease, BMI, PIR, age, and sex.

We performed interaction tests and subgroup analysis, stratified by sex, age, schooling, BMI, income, smoking status, cardiovascular disease, stroke, diabetes, and hypertension to evaluate the consistency of the connection between WWI and both all-cause and CVD mortality in the population as a whole. According to our study, there was an interaction between the relationship between all-cause mortality scores and WWI in different sex subgroups of cancer survivors (P < 0.016). More precisely, the role of male cancer survivors in increasing all-cause mortality was more significant than that of female cancer survivors. Besides, the addition of one WWI unit was associated with a 28% greater probability of mortality from all causes in male patients with cancer. Conversely, among females with cancer, a 2% increase in the probability of all-cause death was linked to every unit increase in WWI related to cancer. There was no interaction between the other subgroup variables for the correlation between WWI and all-cause mortality in cancer patients (p> 0.05 for interaction) (**Figure 4**).

## 4 Discussion

We know no prior research examining the relationship between WWI and cancer patients’ cardiovascular and all-cause deaths. Using a cohort survey with 4,463 representative participants, we discovered an important interaction between WWI with CVD and all-cause mortality of cancer patients. Moreover, we also found a notable sex discrepancy in this association, suggesting that higher WWI in cancer patients, especially men, may increase the death risk. These findings show potential clinical value for assessing WWI in cancer survivors and predicting the risk of cardiovascular mortality, all causes of death.

For decades, academics have been exploring the complex relationship between body composition and survival outcomes in cancer patients. This multifaceted area of research encompasses a wide range of disciplines, from basic physiological rationale to clinical prognostic assessment. Researchers have conducted rigorous analyses to elucidate the potential moderating role of various body composition metrics, including muscle mass and adipose tissue distribution, on long-term survival in cancer populations (18). Utilizing advanced measurement methods and sophisticated statistical frameworks, researchers have progressively revealed the links between these body compositions and key metrics such as cancer progression, treatment outcomes, and ultimately survival (18). For example, a study by Guo et al. revealed the importance of early identification of abnormal changes in a range of key body composition and inflammatory markers in patients with Epithelial Ovarian Cancer (EOC) in predicting response to Olaparib treatment and risk of disease progression (19). Specifically, the study noted that monitoring reductions in visceral adipose tissue index (VATI), skeletal muscle area index (SMI), and body mineral density (BMD), accompanied by elevated neutrophil-to-lymphocyte ratios (NLR), markers of inflammation, provides preliminary evidence for assessing the sensitivity of patients with EOC to Olaparib treatment and the potential risk of disease progression (19). Recently, a study explored the multidimensional associations between various obesity indices and triglyceride glucose index (TyG) with gastrointestinal cancer (GIC) and showed that BMI, waist, and WWI were strongly associated with the prognosis of patients with GIC (20). In addition, numerous investigations exploring the connection between exposure to WWI and death rates from cardiovascular and all-cause causes have been carried out. In one instance, there was a 95% increased risk of cardiac death associated with higher WWI levels (≥11.33) [HR=1.95; 95%CI (1.30-2.93)] and there was a 68% greater possibility of death from all causes [HR=1.68; 95%CI (1.41-2.00)] (21). Nevertheless, our study only considered the association between elevated WWI and all-cause and cardiovascular death among American cancer patients, whereas other studies analyzed all Americans. Another prospective study with 12,447 Chinese participants and a median monitoring of 5.6 years showed a curvilinear favorable correlation between WWI and both all-cause and CVD mortality, while our extended follow-up period of 20 years provided stronger representativeness (22). Furthermore, several other researchers have examined the relationship between cardiovascular disease and WWI. Xiong et al. prospectively analyzed 5,232 Chinese hypertensive individuals and proposed that, in addition to blood pressure management, WWI may be used as an intervention factor for both the avoidance and management of arterial stiffness (23). Similarly, Zhang et al. discovered that increased WWI might have been an independent predictor of heart attack in a longitudinal study involving 25,509 individuals (24). Identical results were obtained from a cohort study including 4,715 Chinese people with hypertension, which suggested that WWI could also independently cause left ventricular hypertrophy (25). These results are consistent with our study’s findings.

Besides, as socioeconomic status rises and people’s dietary habit changes dramatically, a growing public health concern is that up to 35% of American adults are obese (26). This is related to poorer prognosis for several cancer types. Growing preclinical and clinical research suggests a connection between obesity and higher rates of cancer, illness, and death (27, 28). According to a comprehensive study conducted by Eugenia et al. on over 900,000 adult Americans, the present obesity and overweight rates in the country may be responsible for 14% of cancer-related deaths in men and 20% in females. Moreover, an increase in weight is linked to a greater death rate from different cancer kinds in different anatomical regions (4). It is noteworthy that, compared with their slim counterparts, obese patients have worse cancer prognoses and higher rates of recurrence after radiation or chemotherapy (29). These results emphasize how important it is for research efforts to concentrate on comprehending the connection between obesity and cancer prevalence and mortality. Therefore, a critical mechanistic knowledge of the interaction between cancer and overweight may be obtained by clarifying the basic pathogenic characteristics of malfunctioning adipose tissue. Although BMI has traditionally been utilized to classify obesity, the investigation into the association between central obesity and survival has only recently garnered attention, as BMI is considered an unreliable indicator of adipose tissue inflammation (30). WC represents a groundbreaking obesity metric merging the advantages of WC with a reduction in its BMI-dependent correlations, enabling precise identification of central obesity, a condition intricately linked to the increased probability of cardiovascular disease (31). As far as we are aware, this cohort study is the first to look at the relationship between WWI and all-cause and cardiovascular death in cancer patients. While our investigation found significant relative hazards, the observed absolute risk variances were tiny, suggesting that some studies may not be robust enough to identify these effects. Owing to differences in factors that increase vulnerability, or circumstances unique to cancer, early-stage cancer is more common and takes a different shape in obese individuals compared with the general population. Widely studied, WWI is mentioned frequently in research on left ventricular hypertrophy, albuminuria, hyperuricemia, hypertension, and abdominal aortic calcification (32, 33). Regarding central obesity, in particular, WC has become a competitive alternative metric. In many researches, WC has been recommended as a marker of metabolic obesity because of its higher association with visceral fat than BMI (34, 35). Nevertheless, we discovered an adverse association in our study group between WC and all-cause and cardiovascular deaths, highlighting WC’s limits as a predictor that depends on BMI. A causal relationship between WWI and the cancer-prone population may be suggested by the slightly stronger correlation between the two variables, even though the longitudinal approach made it impossible to infer a link of cause and effect between WWI and mortality in the cancer population. In conclusion, WWI is not only simple, economical, and practical to calculate, but also shows excellent ability to predict disease risk, and therefore should be valued by medical practitioners.

Our data indicates that males have a noticeably higher risk of dying from all causes than females. In particular, for every unit rise in WWI, the chance of all-cause death increased by 2% for females and 28% for men who had cancer. A fairly large cohort study in the 1990s that examined the interaction between cancer mortality and BMI showed that men had a higher mortality rate than female for most cancer types (35). These results were comparable to those of our research, and the involvement of oxidative damage, which endures and causes continuous inflammation, and which can then be a mediator of cancer-related events, may contribute to the sex disparity in these findings (36). Studies have shown that hormonal differences between males and females play a crucial role in cancer immunity and the therapeutic response of using the intrinsic immune system to eliminate malignant cells, with estrogens being more immune-stimulating and androgens more immune-suppressing, so that the stronger immune response somehow reduces the cancer mortality rate in female (37). In addition, in men, fat is more likely to accumulate in the abdomen, especially visceral adipose tissue (VAT), while in females it is more distributed in the hips and thighs, as well as subcutaneous adipose tissue (SCAT). However, increased visceral adipose tissue significantly increases insulin resistance, producing adverse metabolic effects that can increase cancer mortality (38). Finally, differences in sex-specific behaviors (e.g. smoking, excessive alcohol consumption, poor dietary habits, etc.) as well as social pressures and mental health problems faced by men may combine to contribute to higher cancer mortality rates in males than in females.

Obesity is caused by a disparity between energy consumption and expenditure. This causes fat tissue to accumulate abnormally over time. There has been a link established over the last twenty years between being overweight and an increased risk of cancer incidence and death. It is estimated that being overweight accounts for 14% of cancer deaths in males and 20% in females (5). Comprehensive clinical and epidemiological evidence also supports the connection between obesity and certain cancers. There may be alternative reasons for the higher association found between WWI and all-cause and cardiovascular death among cancer patients. Firstly, elevated WWI is indicative of dysfunction in adipose tissue, which may lead to increased fat accumulation and inflammation in the body, changes that in turn promote insulin resistance, ultimately accelerating the growth of tumor cells and elevating cancer mortality (39). Secondly, being overweight is associated with persistent subclinical inflammation, aberrant steroid manufacture, and metabolic dysregulation, all of which are linked to the growth and metastasis of malignancies (40). A recent study by Arendt et al. found that obesity creates a chronic inflammatory environment that promotes collaboration between macrophages and adipocytes through the IL-1β/CCL2/CXCL12 signaling pathway, leading to cancer cell growth and metastasis and accelerating the development of breast cancer in obese individuals (41). Thirdly, adipose tissue, as a neuroendocrine organ, can influence obesity in other parts of the body through the release of free fat, peptide hormones, and steroid-related hormones. Through a variety of mechanisms, such as altered sex hormone metabolic processes, increased adipokine synthesis, enhanced activation of insulin-dependent signaling pathways, and changes in the composition of the gut microbial community, adipose tissue increases the risk of cancer development (42). In addition, growth factors like insulin-like growth factor (IGF) and insulin receptors, which promote cell proliferation and inhibit apoptosis, will also contribute to tumor development in the future (43). Furthermore, obesity promotes increased fat deposition in adipocytes, which makes it easier for aromatase activity to convert androgens to estrogen, increasing the risk of breast cancer (44). Finally, studies show that the cholesterol metabolite 27-hydroxycholesterol (27HC) interacts with the Liver X Receptor (LXR) and the hormone receptor, thereby changing the expression of genes that react to estrogen and raising the risk of cancer. Obese individuals frequently exhibit elevated cholesterol levels, such as hypercholesterolemia (45). Studies have shown that 27HC increases tumor growth and metastasis in mouse models. Additionally, they discovered that more aggressive tumors had higher expression of cytochrome P450 oxidase, or CYP27A1, which is responsible for turning cholesterol to 27HC, than did less aggressive tumors (46). This suggests that statins (which lower cholesterol) and inhibitors of CYP27A1 may become new cancer treatment strategies (47).

Based on the results of our study, we suggest that cancer survivors with higher mortality rates can be identified by WWI in the future and appropriate interventions can be guided accordingly. Firstly, during routine follow-up of cancer survivors, their weight and waist circumference should be measured regularly and WWI should be calculated to monitor the development of their health status by comparing changes in the individual indices. Particular attention needs to be paid to those individuals whose WWI is significantly elevated or consistently in the high-risk zone. Secondly, for the identified high-risk groups, we should formulate personalized intervention plans, including nutritional counseling, exercise guidance, and lifestyle modification, to help cancer survivors maintain a healthy WWI. In addition, the effectiveness of the interventions should be assessed regularly, with a focus on the changes in WWI, and the intervention plans should be adjusted on time according to the assessment results, to ensure their relevance and effectiveness. Finally, during the intervention process, attention should also be paid to other risk factors, such as age, sex, cancer type, and treatment history, while maintaining ongoing monitoring and evaluation, which is key to ensuring the effectiveness of the intervention.

The strengths of this study are, first, that it is based on NHANES data, which ensures adequate sample size, reliable mortality data, and a long follow-up period. Second, we conducted careful subgroup analyses, and the complexity of the NHANES population-based survey was fully taken into account in the statistical analyses. Our investigation focused on the association between WWI and all-cause and cardiovascular mortality in cancer survivors, as this could provide stronger evidence for a causal relationship between these two events. However, there are some shortcomings in our investigation, firstly although adjustments were made for several factors, residual confounders could not be completely avoided in an observational study. Secondly, waist circumference and weight were measured at one point in time, which may not capture changes in body composition over time. Longitudinal measurements could provide more insight into the relationship between WWI and mortality. Third, there is a potential bias in the data due to self-reporting of cancer diagnosis and type. Self-reported information is susceptible to recall bias and misclassification, which can affect the reliability of study results. Last, the study’s cohort approach has inherent limitations, such as weight loss tendencies in advanced disease stages, incomplete cancer staging information at the time of questionnaire administration, and a small number of deaths. Therefore, we will conduct more extensive studies in our future research, including clinical trials.

## 5 Conclusion

The results suggest a possible association between a higher risk of cardiovascular death and all-cause death among cancer patients and heightened WWI. In addition, each unit increase in WWI was associated with significantly higher all-cause mortality in male cancer patients than in female cancer patients. Our findings emphasize the importance of obesity monitoring for all cancer survivors to lower complications and improve patient survival and quality of life. This finding may provide future opportunities to identify cancer survivors at higher risk of death, potentially guiding targeted interventions for obesity health management. Next, we plan to conduct additional high-quality prospective studies in our future research, to confirm our results and benefit future studies in related areas.

## Data Availability

The raw data supporting the conclusions of this article will be made available by the authors, without undue reservation.
